# A Comprehensive Assessment of Hip Damage in Ankylosing Spondylitis, Especially Early Features

**DOI:** 10.3389/fimmu.2021.668969

**Published:** 2021-03-24

**Authors:** Qing Han, Zhaohui Zheng, Kui Zhang, Jin Ding, Xenofon Baraliakos, Ping Zhu

**Affiliations:** ^1^ Department of Clinical Immunology, PLA Specialized Research Institute of Rheumatology & Immunology, Xijing Hospital, Fourth Military Medical University, Xi’an, China; ^2^ National Translational Science Center for Molecular Medicine, Xi’an, China; ^3^ Rheumazentrum Ruhrgebiet Herne, Ruhr-University Bochum, Herne, Germany

**Keywords:** ankylosing spondylitis, hip, MRI, X-ray, Harris score

## Abstract

Ankylosing spondylitis (AS) is most common in adolescents and the ultimate result is disability, which places a huge burden on patients and society. Therefore, the key to improve the prognosis of AS is the early diagnosis of hip injury. To examine if AS patients whose hip pain is either absent or minimal might already have observable MRI and X-ray hip changes. Clinical and imaging hip data were systematically analyzed in 200 healthy controls (HC) and 300 AS with varying degrees of hip pain. Forty-four patients with early hip osteoarthritis (OA) served as positive imaging controls. In MRI images, BME lesions in the STIR sequence were much more frequent in AS (62%) compared to HC (2%) (p < 0.0001). Most importantly, 42% of AS with no or minimal hip pain had one or more MRI lesions. This was much more frequent compared to the 2% in HC (p < 0.05). These lesions in AS were observed singly or in combination in the trochanters (8%), femoral heads (12%), and acetabula (13%). Parallel finding that X-ray changes were present in patients with minimal or no hip pain was also observed with X-ray. Based on the normal hip width of HC, joint space narrowing was observed in 94.3% of the entire AS cohort, and importantly 56.7% of AS patients with no or mild hip pain. In these latter patients, functional activities of the hips such as walking were normal. At least 40% of AS patients with minimal or no hip pain might already show MRI and X-ray changes.

## Introduction

Axial spondyloarthritis consists of two groups of entities, ankylosing spondylitis (AS), also known as radiographic axial spondyloarthritis, and non-radiographic axial spondyloarthritis (nr-axSpA). Radiographic sacroiliitis is present in AS but not in nr-axSpA (1). In the majority of ethnic groups, many AS patients carry the HLA-B27 gene (1–3). Other than pain, the two most common disabling features are bridging of vertebral syndesmophytes and hip destruction (4–6). The disease activity in many patients can be controlled by a TNF inhibitor, an IL-17 inhibitor, or a Janus kinase inhibitor (4, 7, 8). Not all patients develop syndesmophytes or hip destruction (9). It is commonly thought that early treatment can prevent syndesmophyte formation (10, 11). However, prevention of progression has not been studied for hip destruction. Such studies need markers that might identify early hip disease.

Hip lesions have been reported in 25 to 35 percent of AS patients (12, 13). The typical symptom is groin pain. As a whole, hip lesions may be more severe in patients with early age onset, highly active axial, and entheseal diseases (14, 15). Very few imaging studies have been done with the aim of identifying early hip lesions. By the time a patient develops even moderate hip pain, the destructions are usually extensive (12). By that time, due to the lack of alternate treatment, hip arthroplasty is often the only effective therapy (12). Almost 90 percent of patients will experience pain relief and improved ranges of motion from hip arthroplasty. Ninety percent survived the replaced hip for 10 years, and 72 percent for 15 years (16, 17). Nevertheless, in theory, a patient would prefer using medications to arrest the progression of destruction before it requires arthroplasty.

One of the major objectives of this project is to examine if patients with minimal or no hip pain might already have X-ray and MRI changes. Investigators can then test if the progression of arthritis can be arrested by more aggressive medical therapies.

## Methods

### Study Design and Participants

Two hundred healthy control (HC), 300 outpatients with AS with or without hip pain, and 44 patients with early OA of hips were recruited from the clinics of the Department of Clinical Rheumatology at Xijing Hospital, Xi’an, China. All AS patients fulfilled the 1984 modified New York criteria. Their spondylitis was regarded as being clinically active. None of them were on biologics at the time of evaluation. We follow three steps. The first step is to identify among a list of clinical parameters, the one which by itself is most useful for assessing hip involvement. The second is to identify X-ray parameters that might appear in early hip involvement. The third is to identify corresponding MRI changes. As for comparisons, we also investigate patients with moderate and severe hip pain. For positive controls, we used early hip osteoarthritis.

None of AS patients had experienced hip injury or showed non-Spondyloarthritis causes of hip arthritis. Patient with psoriasis or inflammatory bowel disease were excluded from the study. The study and the informed consent have been approved by the ethics committee of the Xijing Hospital of Fourth Military Medical University (ID: 20110303-7).

### Radiography

X-rays were taken with subjects in the supine position with the targeted hip(s) at the center of focus. They were instructed to extend their lower limbs, and to rotate them so that the two big toes would touch one another. The upper margin of the image included the superior iliac crests. The lower margin included the upper third of the femurs. Quantitative hip joint width (HJW) measurements were assessed on images in a DICOM viewer. Three sites were measured: superomedial, superolateral, and the point of narrowest part of each hip space. For each patient, the narrowest side of the three measurements was used for comparison to other patients. The team of readers consisted of two radiologists and two rheumatologists. Readers were blinded to the clinical data. In our own cohort, the X-rays of the hips of OA patients were assessed systematically for the following three features: osteophytes, subchondral sclerosis and femoral head deformity.

### MRI

MRI examinations were performed using standard protocols for T1W1 and STIR sequences using a 1.5-T machine (Magnetom Aera; Siemens Medical Solutions, Erlangen, Germany). Patients were placed supine with the hip joint in a neutral position. The parameters for T1-weighted images were TR/TE 715/9.5ms, for coronal short-tau inversion recovery (STIR), repetition time was 3550, echo time 51, and inversion time 145ms. Slice thickness was 4 mm. Both hips were included in the same image. Formal readings were preceded by a learning session. In the learning session, MRIs of a randomly selected subset of subjects were read separately by the four experienced readers. We divided the intense STIR signals into the following categories: subchondral acetabular, subchondral femoral head, cysts in acetabula, cysts in the femoral heads, enthesitis at greater trochanter, enthesitis at lesser trochanter. The readers were blinded to the clinical data. Afterwards, the four readers reviewed their scores together and discussed about the discrepancies. This learning session was followed by formal reading of all MRI. Disparities were reviewed together to arrive at a consensus.

### Statistical Analyses

Analyses were performed using the SPSS 19.0 software (IBM, Armonk, NY, USA). Because the distribution was non-parametric, the Mann-Whitney U test was used for intra-group comparisons. In descriptive analysis, quantitative parameters were expressed as means and standard deviations. Qualitative parameters were expressed as percentages. Principal component analysis (PCA) was used to assess the percent contribution of each of different parameter. Kruskal-Wallis test was used for comparing samples of different sample sizes. Spearman’s rank correlation test was used to evaluate the degree of correlation. The threshold statistic significance was set at p < 0.05.

## Results

### Demographic

In **Table 1**, we show the demographic of the 300 AS patients, 44 OA patients and 200 healthy control. Eighty six percent of AS patients were HLA-B27 positive. The mean age of the AS group is not statistically different from the healthy control but less than the OA group (p < 0.05 comparing mean ages between AS and OA).

**Table 1:** Demographic features and pretreatment values for evaluation parameters of groups.

Characteristics (mean ± SD or %)	HC group	OA group	AS group
number of individuals	200	44	300
Age in years	29.9 ± 10.45	69.9 ± 5.54	25.8 ± 10.32
Male	73%	54%	82%
Duration (Month)	NA	NA	67.38 ± 65.39
Harris Hip Score (100 = best health)	94.49 ± 6.59	68.4 ± 6.33	77.82 ± 8.82
Hip pain score (44 = best health)	0	39.19 ± 6.65	24.95 ± 5.57
Flexion (degree)	132.8 ± 16.17	129.1 ± 22.3	125.31 ± 2.67
Abduction (degree)	29.16 ± 6.62	28.24 ± 6.15	26.19 ± 6.62
Extorsion (degree)	25.62 ± 0.61	25.5 ± 3.45	13.61 ± 6.61
Adduction (degree)	24.82 ± 2.46	22.5 ± 3.43	23.8 ± 5.45
Walking distance (m)	>1000	634 ± 256.6	521.2 ± 341.8
Function (0 = normal)	NA	32.78 ± 6.12	38.05 ± 5.11
BASDAI (0-10)	NA	NA	5.69 ± 1.58
BASFI (0-100)	NA	NA	37.48 ± 18.78
BASMI (0-10)	NA	NA	1.26 ± 1.98
Patient Global (0-10)	NA	NA	8.02 ± 1.25
CRP (higher than normal)	NA	NA	71%
ESR (higher than normal)	NA	NA	78%
HLA-B27 positive	NA	NA	86%

HC, Health control; OA, Osteoarthritis; AS, Ankylosing Spondylitis; BASDAI, Bath Ankylosing Spondylitis Disease Activity Index; BASFI, Bath Ankylosing Spondylitis Functional Index; BASMI, Bath Ankylosing Spondylitis Metrology Index; CRP, C-reactive protein (normal <0.8mg/dl); ESR, Erythrocyte Sedimentation Rate (normal Male<15mm/h, Female<20mm/h). Numbers are in means ± SD. NA, not available.

### Identifying the Most Useful Clinical Parameter to Assess Hip Involvement in AS

We first used the parameters in the Harris Score as a clinical tool to assess hip joint disease activity and function. These parameters included hip pain, three separate hip ranges of motion (flexion, external and internal rotation), function, and walking distance. The Harris scores of both AS (77.5 ± 8.7) and OA (81.2 ± 6.4) were significantly lower than those of healthy normal controls (94.1 ± 3.6) (p < 0.0001). In AS, correlation analysis showed that among the variables in the Harris score, a strong correlation was observed only between pain and walking distance (r = 0.7, p < 0.0001). This showed that each parameter was relatively independent of the others. In AS, Harris score did not correlate with age, disease duration, BASDAI, BASMI, BASFI, ASDAS, CRP or ESR (p > 0.5). The lack of correlation to those parameters indicated that for AS, the Harris score was an independent evaluation tool. We used PCA to calculate the percent of contribution of each of the variable within the Harris score to the final score. PCA showed that pain accounted for at least 50% of the total variables. The contribution of the other parameters was relatively even and much less than that from pain. Harris Score divided the intensity of pain into three categories: minimal/none, moderate and severe. For hip pain, in our cohort, 33% of patients reported minimal/no pain, while 28% reported moderate pain, and 39% severe pain. In the OA cohort, 73% reported minimal/no pain, while 21% reported moderate pain, and 6% severe pain.

### Positive Controls for X-ray and MRI Changes Using OA Images

Results are of X-ray features of the hips were tabulated in **Table 2**. In the 200 subjects in the HC group, osteophytes subchondral sclerosis and femoral head deformity were present in 1%, 2% and 0% respectively. For the 44 OA patients, those features were much more frequently at 29%, 87% and 32% respectively (p values of each ranged from < 0.001 to <0.0001). The joint width of hips in our OA cohort varied considerably. The mean and SD were 3.2 ± 0.7 mm. The mean was less than that of the 4.8 ± 0.74 mm of HC (p < 0.001). When analyzed by regression analysis, there was a small degree of correlation between the joint width and degree of pain (r = 0.408, p = 0.006). As expected, the mean joint width was the smallest in the group with severe pain.

**Table 2:** Statistical comparison of X-ray and MRI features of hips in HC, AS and OA subjects.

X ray	Number of individuals	Joint width (mm)	Osteophytes	Subchondral sclerosis	Femoral head deformity			
HC	200	4.8 ± 0.74	1%	2%	0			
AS	300	1.5 ± 0.8*#	2%#	22%*#	1%#			
OA	44	3.2 ± 0.7*#	29%*#	87%*#	32%*#			
MRI	Number of individuals	BME lesions in trochanters	Superficial BME lesions in femoral heads	Superficial BME lesions in acetabula	Deep BME lesions in femoral heads	Deep BME lesions in acetabula	Cysts in femoral heads	Cysts in acetabula
HC	200	0%	1%	1%	0%	0%	0%	0%
AS	300	14%*#	56%*#	38%*#	29%*#	13%*#	0%#	10%*#
OA	44	0%#	30%*#	2%#	4%#	1%#	14%*#	0%#

HC, Healthy control; AS, Ankylosing Spondylitis; OA, Osteoarthritis; BME, bone marrow edema. Numbers in mean ± SD. statistically different from healthy control, p < 0.05~0.0001; # statistically different between AS and OA, p < 0.05~0.0001. All MRI lesions were observed with the STIR sequences.

We systematically evaluated the STIR images of the OA hips for BME lesions in trochanters, superficial subchondral BME lesions in the femoral heads, superficial subchondral BME lesions in the acetabula, deep subchondral BME lesions in the femoral heads, deep subchondral BME lesions in the acetabula, cysts in the femoral heads, and cysts in the acetabula. The lesions designated as cystic were distinguished from the BME lesions by their very distinct outline. Results are tabulated in **Table 2**. In our OA hip cohort, there was no BME lesion in the trochanters or cysts in the acetabula. The only STIR lesions that distinguished OA from HC were superficial subchondral BME lesions in the femoral heads and cysts in the femoral heads. We then searched for similar appearing BME and cystic lesions in AS.

### X-ray and MRI in AS Patients

**Figure 1:**
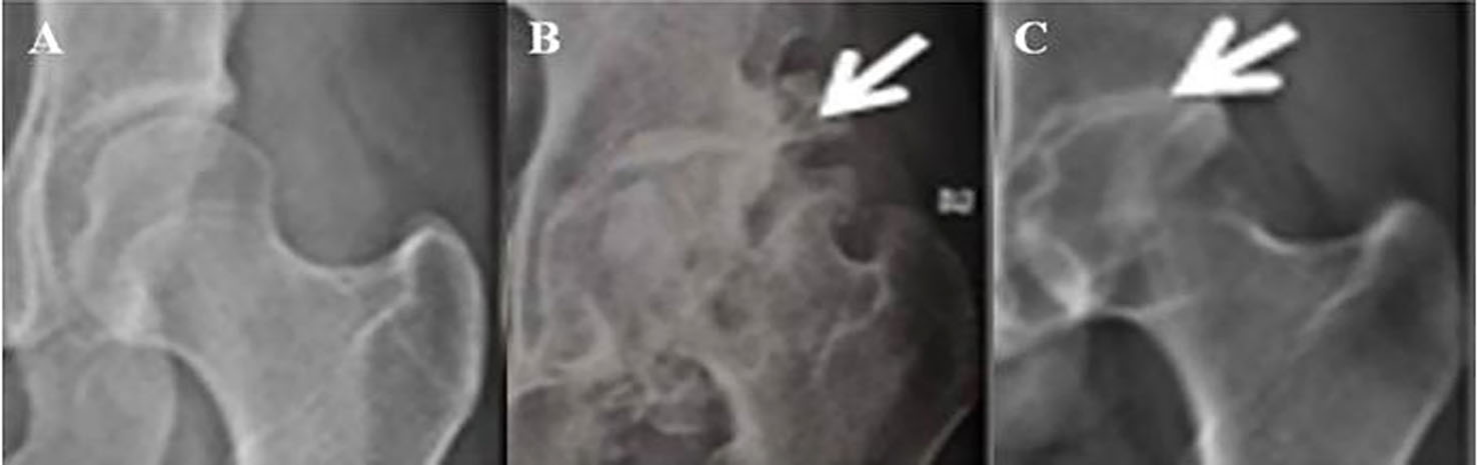
X-rays of hips in a healthy control, OA and AS subject. **(A)** a healthy control subject; **(B)** an OA patient. White arrow points at an osteophyte; **(C)** an AS patients. White arrow points at narrowing of joint space.

Hip X-rays were systematically evaluated to determine the following characteristics: osteophytes, subchondral sclerosis and femoral head deformity. The upper half of **Table 2** compares X-ray features in HC and AS subjects. Compared with HC subjects, only subchondral sclerosis appeared to be more frequent in AS than that of the control group (22% in AS versus 2% in control, p = 0.008). In addition to the above 3 parameters, we also measured joint widths of the involved hips. The mean and standard deviations of joint width in the healthy control group were 4.8 ± 0.74 mm. The range was 3.0 to 6.6 mm. Using the same method of measurement, the mean and standard deviation of the affected AS hips were much lower at 1.5 ± 0.8 mm (p < 0.00001). Regression analysis showed that there was a high degree of a statistical relationship between joint width and pain (r = 0.81, p < 0.001). If the lower limit of normal is set at 3.89 mm, 94.3% of the involved hips in AS were below the normal threshold regardless of the presence or absence of pain. Even more striking, 56.7% of AS patients with no or mild hip pain showed a narrowing of hip-width. We also compared the hip joint width of AS patients who have minimal/no hip pain (3.3 ± 0.66 mm) to mild hip pain (2.15 ± 0.63 mm), to moderate hip pain (1.65 ± 0.26 mm), and severe hip pain (0.93 ± 0.56 mm). The mean hip joint width of each group was significantly different from one another (p < 0.01) (**Figure 1**).

In the healthy controls, the percent of hips with BME lesions in trochanters, superficial subchondral BME lesions in the femoral heads, deep subchondral BME lesions in the femoral heads, superficial subchondral BME lesions in the acetabula, deep subchondral BME lesions in the acetabula, cysts in the femoral heads, and cysts in the acetabula were observed in only 0%, 1%, 1%, 0%, 0%, 0% and 0%, of all the hips. The corresponding values for AS were 14%, 56%, 38%, 29%, 13%, 0%and 10% respectively (**Table 3**).

**Table 3:** Statistical comparison of MRI features in different hip pain subgroups.

AS -hip pain	Number of individuals	BME lesions in trochanters	Superficial BME lesions in femoral heads	Superficial BME lesions in acetabula	Deep BME lesions in femoral heads	Deep BME lesions in acetabula	Cysts in femoral heads	Cysts in acetabula
Minimal/mild ^a^	103	8%*	12%*	13%*	5%*	6%*	0%	4%*
Moderate	142	3%	13%	11%	3%	1%	0%	1%
Severe	55	1%	31%#	12%	21%#	6%	0%	5%

HC, Healthy control; AS, Ankylosing Spondylitis; OA, Osteoarthritis; BME, bone marrow edema. * indicates that the particular minimum/mild hip pain group value is statistically higher than the healthy control group; # represents statistically significant difference between the severe hip pain group and the minimal/mild hip pain; ^a^42% of this group has at least one MRI sign. All MRI lesions were observed with the STIR sequences.

**Figure 2:**
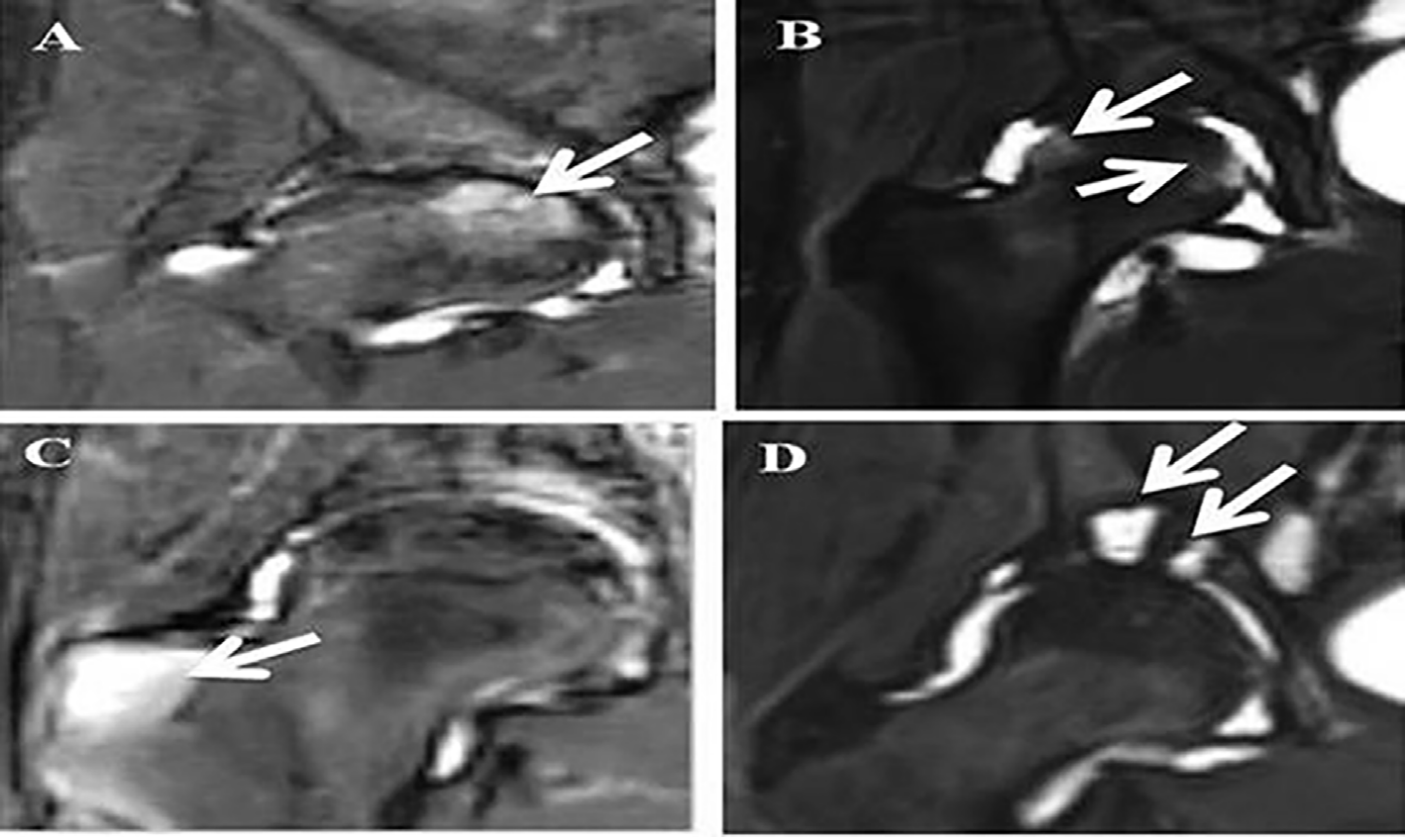
MRI STIR images of hips in AS patients. Legends: **(A)** White arrow points at a deep BME lesion in the femoral head; **(B)** White arrows point at superficial BME lesions in the femoral head; **(C)** White arrow points at a BME lesion in the greater trochanter; **(D)** White arrow points at two cysts in the acetabulum.

All parameters except cysts in the femoral head were statistically more frequent in AS than those in the HC group (p values ranged from < 0.05 to <0.0001). Interestingly, some of the BME lesions appeared to extend inward from the areas where the femoral heads engaged the labrum (**Figure 2**).

When we confined our focus on the group of AS patients with minimal or no pain, we observed three differences from the HC group. Firstly, there were 8% in AS with BME lesions in trochanters. Secondly, 36% of AS showed subchondral BME lesions, and 4% of AS showed cystic lesions. All three MRI lesions were practically non-existent in the HC group. Overall, 42% of those with minimal or no pain in the hips showed one or more MRI lesions. Specifically, we did not observe any fat metaplasia.

### X-ray and MRI Changes and Clinical Parameters in the Group With Minimal or No Hip Pain

For X-ray features, we first focus on joint width. The lowest width in the HC group is 3.0 mm. If we consider the lowest 95% Percentile, the lowest limit is 4.0 mm. In the group of patients with minimal/no hip pain, 44% have width less than 3.0 mm and 88% have width less than 4.0 mm. When we examine for MRI changes, 58% show at least one MRI change, 39% show more than one MRI changes. The MRI changes and clinical parameters are shown in the accompanying **Table 4**.

**Table 4:** Comparison of X-ray and MRI changes and clinical parameters in no or minimal hip pain group.

Parameters	Percentage
X-ray width less than 3 mm (%)	44
X-ray width less than 3.85mm (%)	82
X-ray width less than 4 mm (%)	88
at least one MRI change(%)	58
more than one MRI changes(%)	39
**MRI features:**	
Superficial subchondral BME lesions in the femoral heads (%)	53
Superficial subchondral BME lesions in the acetabula (%)	31
Deep subchondral BME lesions in the acetabula(%)	17
Deep subchondral BME lesions in the femoral heads(%)	14
BME lesions in trochanters(%)	22
Cysts in the acetabula(%)	11
BASDAI ≧4.0(%)	26
High CRP^a^(%)	25
High ESR^b^(%)	22

BME, bone marrow edema; BASDAI, Bath Ankylosing Spondylitis Disease Activity Index; CRP, C-reactive protein (<0.8 mg/dl); ESR, Erythrocyte Sedimentation Rate (Male<15 mm/h, Female<20 mm/h).^ a^CRP higher than normal; ^b^ESR higher than normal.

## Discussion

AS places a huge burden on both patients and society. Inflammation of hip joint can eventually lead to bone damage, which result in decreased physical function and psychological disorders such as anxiety and depression. This further results in the need for help at work, increased sick leave and even incapacity. In addition, patients still need to spend a high amount of medical resources. It is important to note that the burden of disease increases with the course of the disease. Acute inflammatory findings on MRI can predict bone structure changes on late radiographs. Patients with early, typical presentation of AS may not have definite X-ray abnormalities, but further MRI examination may reveal early inflammation. Therefore, early diagnosis and treatment are crucial to improve prognosis.

Hip involvement has been recognized to be present in 19% to 47% of AS patients in several studies (18, 19). The largest of these is a composite of three data sets consisting of more than 2,000 AS patients. In these AS cohorts, hip involvement was present in about a third to a quarter of the patients. As in all studies, hip involvement is associated with a higher degree of functional impairment. Therefore diagnosis, especially early diagnosis should be an important part of AS management. In the above study, hip involvement was defined as either of three parameters: Clinical assessment, radiographic assessment, and need for hip replacement. It is not clear what are the most sensitive methods for either clinical or radiographic assessment. One recent study of 60 AS patients assessed the usefulness of the BASRI-score, which grades the radiographic severity from 0 to 4 using four separate parameters (9). However, the study did not use HC or reveal which particular of the four radiographic parameters was the most useful for identifying early hip involvement.

We know from sacroiliitis that MRI has the potential of showing pathologies before obvious radiographic changes. Two studies are promising in showing that more than 70% of AS patients with clinical hip involvement showed MRI abnormalities (20, 21). The changes they observed were joint effusion, bone marrow edema, and bone erosions. However, there were no HC in their studies. Based on numerous studies on MRI of the sacroiliac joints and the spine, unless MRI of HC for false positives are taken into account, it is impossible to assess which MRI changes of the hips can provide a diagnosis for preclinical and early clinical hip involvements.

We need to examine if patients with minimal or no hip pain might already have MRI changes. To ensure that we could identify MRI lesions, we first used patients with OA as positive controls. This is because MRI changes have been well described in OA, and typical examples are publically available. We then proceeded to study AS. In MRI images, BME lesions in the STIR sequence were much more frequent in AS (62%) compared to HC (2%) (p < 0.05 to 0.0001). Most importantly, 42% of AS with minimal or no hip pain had one or more MRI lesions. This was much more frequent compared to HC (p < 0.05). These lesions were observed singly or in combination in the trochanters (8%), femoral heads (12%)and acetabula (13%). For X-ray images, the most significant finding is that there was a high correlation between the joint width of the involved hips with hip pain (r = 0.81, p < 0.001). Using the HC to set the lower limit of normal, narrowing was observed in 94.3% of the entire AS cohort, and importantly 56.7% of AS patients with no or mild hip pain. To our knowledge, narrowing of hip joint width with minimal or no hip pain has not been reported before.

In AS, overall disease activity in many patients can be controlled by TNF inhibitors, IL-17 inhibitors or Janus Kinase inhibitors. For TNF inhibitors, early treatment is often considered able to retard radiological vertebral progression. However, this has not been studied in hip studies. Part of the difficulty is that there are no markers for early hip disease. The present study offers several promising parameter for future investigation. Since it is not practical to subject every AS patients to MRI of the hips, we studied the X-ray changes in detail. Our results suggest that some AS patients with no or minimal pain might already show joint width narrowing on X-rays. Our results also showed that some of them might have normal acute phase reactants and low disease activity. In clinical practice, it would be reasonable to submit those patients with narrowed joint width to MRI evaluation of their hip joints. This indicated the joint width is a potentially very cost-effective objective imaging parameter to assess for both the presence as well the severity of hip involvement in AS.

The present MRI study adds to the existing information because the cohort is much larger, and because we use healthy control as negative control, and OA as positive control. Further, we categorize patients into groups of symptoms severity.

### Limitations

The present study has several shortcomings. The definitions of X-ray and MRI lesions were arbitrarily chosen because there is no standardization available. The method of measuring joint width might vary depending on how the X-rays are taken. In our study, all X-rays were taken with the same protocol. The presence of joint width narrowing in AS is not totally unexpected. What is unexpected is that there is considerable number of patients with joint width narrowing even when they have minimal or no hip pain. Similarly, the presence of MRI changes described is not unexpected. What is unexpected is that they can be present in patients with minimal or no hip pain. Another shortcoming is that we did not detect significant MRI changes with the T1 sequence. This might be partly because we were using a low-resolution machine. Another shortcoming is that the OA we report were used as positive controls. Our OA cohort consisted those with early OA. Any differences between the AS and the OA we observed do not reflect general differences between the two diseases. The most important shortcoming is that this is a cross-sectional study. Any changes we observed might theoretically be transient. However, the study provided parameters which we can use in the future in longitudinal studies.

## Conclusion

Hip involvement, which occurs in at least 20% of patients with AS, is associated with a high degree of disability. In current clinical practice, accurate diagnosis relies on X-ray, which is usually used only when there is hip pain and probably misses the early cases. This study discovers that AS patients with minimal or no hip pain might already have silent X-ray and MRI changes. Studies are needed to test whether AS patients with X-ray and MRI signs of early hip arthritis should be treated with aggressive medical therapies. The current research provides those X-ray and MRI parameters.

## Data Availability Statement

The raw data supporting the conclusions of this article will be made available by the authors, without undue reservation.

## Ethics Statement

The study and the informed consent have been approved by the ethics committee of Xijing Hospital of Fourth Military Medical University (ID: 20110303-7). The patients/participants provided their written informed consent to participate in this study. Written informed consent was obtained from the individual(s) for the publication of any potentially identifiable images or data included in this article.

## Author Contributions

QH and PZ contributed to the conception of the work and completed the first draft and final version of the manuscript. QH, ZZ, KZ, JD, and XB contributed to the design of the work. QH, ZZ, KZ, JD, and XB contributed to the data acquisition and analysis. QH, ZZ, KZ, JD, XB, and PZ contributed to interpretation of data. All authors were involved in the manuscript revision and agreed with final approval of the version, and ensured the accuracy of investigation.

## Funding

This study was funded by the National Basic Research Program of China (2015CB553704) and the National Nature Science Foundation Key Research Project of China (2017YFC0909002).

## Conflict of Interest

The authors declare that the research was conducted in the absence of any commercial or financial relationships that could be construed as a potential conflict of interest.
